# Polyoxometalates as components of supramolecular assemblies

**DOI:** 10.1039/c9sc00979e

**Published:** 2019-03-22

**Authors:** Maria Stuckart, Kirill Yu. Monakhov

**Affiliations:** a Institut für Anorganische Chemie , RWTH Aachen University , Landoltweg 1 , 52074 Aachen , Germany; b Jülich-Aachen Research Alliance (JARA-FIT) , Peter Grünberg Institute (PGI-6) , Forschungszentrum Jülich GmbH , Wilhelm-Johnen-Straße , 52425 Jülich , Germany; c Leibniz Institute of Surface Engineering (IOM) , Permoserstr. 15 , 04318 Leipzig , Germany . Email: kirill.monakhov@iom-leipzig.de

## Abstract

The non-covalent interaction of polyoxometalates (POMs) with inorganic- or organic-based moieties affords hybrid assemblies with specific physicochemical properties that are of high interest for both fundamental and applied studies, including the discovery of conceptually new compounds and unveiling the impact of their intra-supramolecular relationships on the fields of catalysis, molecular electronics, energy storage and medicine. This minireview summarises the recent advances in the synthetic strategies towards the formation of such non-covalent POM-loaded assemblies, shedding light on their key properties and the currently investigated applications. Four main emerging categories according to the nature of the conjugate are described: (i) POMs in metal–organic frameworks, (ii) POMs merged with cationic metal complexes, (iii) architectures generated with solely POM units and (iv) POMs assembled with organic molecular networks.

## Abstract 2

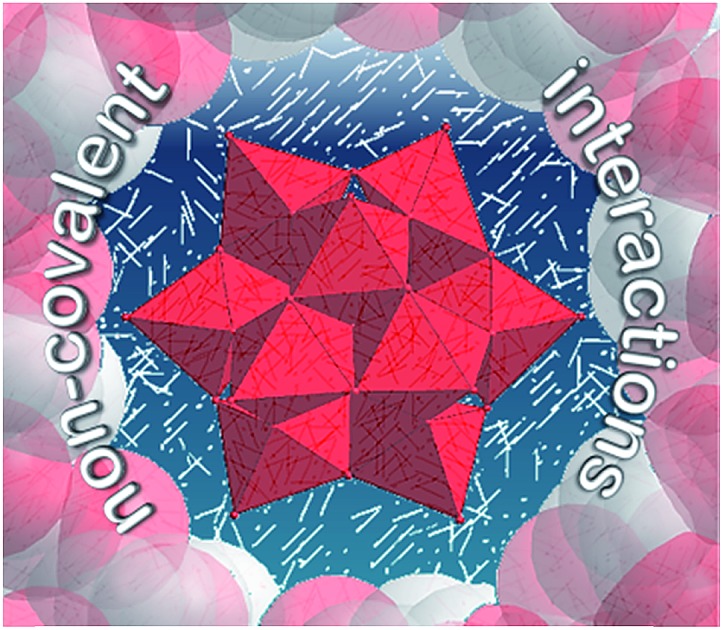
The non-covalent interactions between polyoxometalates and inorganic- and organic-based moieties give rise to functional nanoassemblies with diverse potential in applied science.

## 1 Introduction

Advancing the miniaturisation, compatibility, performance and energy efficiency of electronic, magnetic, sensing and catalytic devices requires a continuous search for synthetic approaches to hybrid molecular objects with well-defined, controllable and processable nanoscale structures.^1^ The utilisation of metal–oxo clusters or so-called polyoxometalates (POMs), in particular, molybdenum-, tungsten- and vanadium-based polyoxoanionic species, as precursors is one of the promising directions for the synthesis of nanostructured materials, which has gained increasing attention from experimental communities.^2^–^15^ The interest in POMs is triggered by a large variety of their stable structural motifs that are capable of undergoing (i) chemical modification by main-group elements^16^ and/or organic and organometallic moieties,^6^,^7^ (ii) alteration of intrinsic magnetic properties, *e.g.* by encapsulation of guest species3b–d or magnetic functionalisation by other heterometallic ions3a,^17^ and (iii) reversible redox processes at different pH.^4^,^18^

In comparison with the extensively studied covalent attachment of various organic and inorganic functionalities to POM skeletons,^6^,^7^ the ionic and other non-covalent interactions (hydrogen bonding, van der Waals forces, *etc.*) between polyoxoanions themselves and between POMs and other types of molecular systems have remained considerably under-investigated over the past years. This is despite the obvious fact that the negatively charged POMs provide a vast platform for the investigation of their electrostatic interactions with a large variety of cationic species. The recent development of straightforward preparation routes to hybrid materials bearing POM units through supramolecular contacts prompted experimentalists in many scientific fields to take a closer look at the properties and reactivity of these molecular nanoassemblies.^14^ Their potential for application in the fields of catalysis,^19^ biochemistry,^20^ energy storage^21^ and materials science and technology^22^–^24^ has been extensively explored.

One of the significant specifics inherent to such supramolecular assemblies is that in most cases their underlying formation does not significantly influence the structural characteristics of POM building units, thus allowing the handling and the addressing of the latter to be comparable to how they would be for individual POM molecules in solution or in the solid phase. Furthermore, the versatility of the physicochemical properties of metal–oxo cluster units and other molecular components of these supramolecular assemblies can be synergistically enhanced, resulting *inter alia* in improved efficiency of the whole hybrid architecture in *e.g.* catalytic reactions^25^ or dye adsorption.^26^

**Fig. 1:**
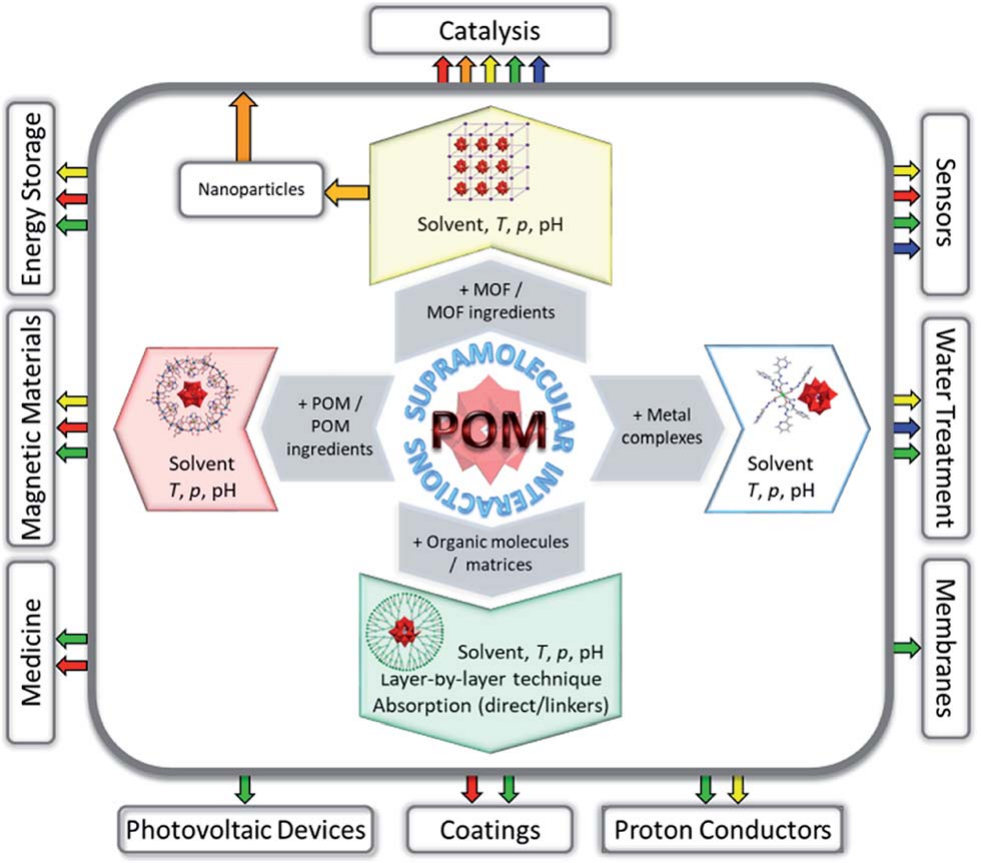
Overview of possible applications reflecting the results published so far for various types of POM-containing supramolecular assemblies.

Non-covalent interactions and networking between POMs and varied molecular systems have so far enabled the convenient isolation of previously elusive polyoxoanions in crystalline form,^27^ stabilisation of hypersensitive magnetic POM species,^28^ increase of the POM surface area with an impact on catalysis,^19^ optimisation of the biocompatibility of polyoxoanions^20^ and uniform arrangement of polyoxoanions on surfaces.^21^,^29^ The synthesis, properties and currently tested areas of application of the most important and interesting representatives of the emerging class of POM-containing supramolecular assemblies (Fig. 1) are described herein. Perspectives for these compounds in the utilisation as constituents of future technology devices are briefly discussed.

## 2 POM-based supramolecular hybrid compounds

### 2.1 POMs in metal–organic frameworks (MOFs)

MOFs are structurally ordered porous materials built from metal-containing nodes that are linked together *via* organic groups.^30^ MOFs arouse substantial interest, in particular, due to the possibility of configuring their skeletons and the content of their pores and of imparting valuable catalytic^31^ or magnetic^32^ properties to assemblies formed on their basis.

The non-covalent grafting of POMs onto MOFs, resulting in hybrid materials hereafter abbreviated as POM@MOFs, is generally realised by impregnation of pre-synthesised MOFs in POM solutions^19^,^33^,^34^ or using solvo- and hydrothermal methods if *in situ* formation of host–guest POM@MOF assemblies can thus be attained.^35^–^39^

A preparation of [Gd(bipyNO)_4_]_3_(W_6_O_19_)_3_(TfO)_3_·solvent (bipyNO = 4,4′-bipyridyl-*N*,*N*′-dioxide; TfO = triflate; solvent = mixture of MeOH and CH_2_Cl_2_) exemplifies the first approach. This hybrid compound was formed due to the exchange of some of the TfO^–^ anions, occupying the cavities of the parent [Gd(bipyNO)_4_]_3_(TfO)_9_·solvent MOF, for [W_6_O_19_]^2–^ polyanions. The results of powder X-ray diffraction (XRD) experiments revealed that in contrast to the POM-free MOF the produced POM@MOF compound preserves its structure even after solvent removal.^34^

**Fig. 2:**
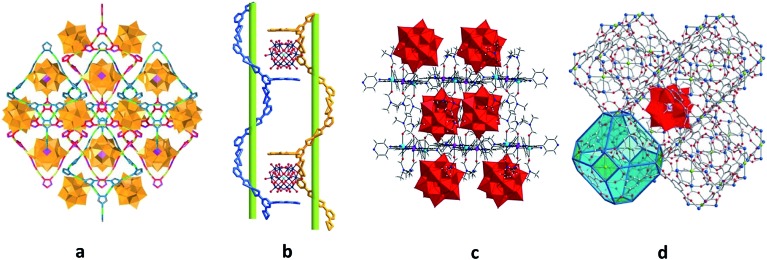
Polyhedral and ball-and-stick representation of POM@MOF structures. (a) The solid-state {[Ag_2_(trz)_2_][Ag_24_(trz)_18_]}[PW_12_O_40_]_2_ segment structure. The two interpenetrating frameworks are differentiated by color for clarity. Each of these two frameworks is built of interlocking cages. Colour code: W, orange; P, pink polyhedra. Reprinted with permission from Springer Nature: ref. 38, Copyright 2010. (b) Schematic representation of the solid-state {[Ce_4_(BINDI)_2_(DMA)_16_]·[SiW_12_O_40_]}_*n*_ segment structure. Colour code: W, blue; Si, cyan; O, red spheres. Adapted from ref. 39 with permission from The Royal Society of Chemistry. (c) The solid-state {[Cd(DMF)_2_Mn(DMF)_2_TPyP](PW_12_O_40_)} segment structure. Colour code: Mo, red polyhedra; Cd, cyan; Mn, pink; N, blue; C, grey; H, light grey; O red spheres. (d) NENU-11. Colour code: Mo, red polyhedra; Cu, blue; P, pink; C, grey; Cl, green; O, red spheres. Reprinted with permission from ref. 36. Copyright 2011 American Chemical Society.

The templating effect of POMs on the MOF self-assembly process^36^,^38^,^39^ exemplifies the second approach. Here, the mixture of MOF constituent elements with pre-synthesised POMs or POM ingredients is treated under solvo- or hydrothermal conditions. Frequently, the formation of the POM-free MOF structure in the absence of the respective POM units was not observed.^36^,^38^ For instance, the generation of a 3D polycatenated structure {[Ag_2_(trz)_2_][Ag_24_(trz)_18_]}[PW_12_O_40_]_2_ (trz = 1,2,4-triazole), which is depicted in Fig. 2a, was possible only with the support of [PW_12_O_40_]^3–^ POM units that are embedded in the pores of the MOF assembly *via* electrostatic interactions as well as weak coordination bonding with silver ions (Ag(MOF)···O(POM) 2.6737 Å). Interestingly, the MOF component of this compound is constructed not by the commonly observed covalently bound building blocks but by the interpenetrating individual, octahedrally shaped {Ag_24_(trz)_18_}^6+^ frames that are reminiscent of chain links.^38^ Another example of a POM-templated MOF is the visible-light-responsive photochromic hybrid [Ce_4_(BINDI)_2_(DMA)_16_]·[SiW_12_O_40_]·3DMA (BINDI = *N*,*N*′-bis(5-isophthalate)-1,4,5,8-naphthalenediimide, DMA = *N*,*N*-dimethylacetamide) in Fig. 2b, where the [SiW_12_O_40_]^4–^ POM units interact with the 1,4,5,8-naphthalenediimide MOF fragments through non-covalent anion–π contacts and C–H···O hydrogen bonds.^39^

Further optimisation of the above-described synthetic approaches towards POM@MOFs is however required, in order to get more control of the self-assembly processes leading to the formation of the materials with desired structures and properties.

**Fig. 3:**
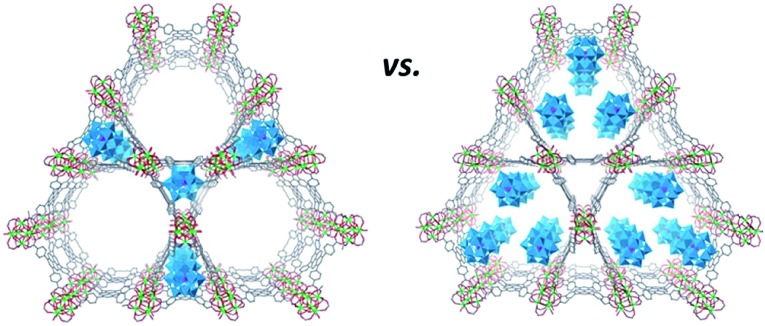
Schematic representation of possible locations of the Keggin-type [PW_12_O_40_]^3–^ units in the NU-1000 MOF. Colour code: W, blue; P, pink polyhedra; Zr, green; P, pink; C, gray; O, red spheres. Adapted from ref. 40 with permission from The Royal Society of Chemistry.

It is noteworthy that the fine-tuning of the POM location in certain MOF cavities could be accomplished in several cases.^33^,^40^ For instance, the possibility of post-synthetically manipulating the position of POM units within the MOF was recently explored for the [PW_12_O_40_]^3–^@NU-1000 system (Fig. 3). The presence of [PW_12_O_40_]^3–^ in the microporous channels of the MOF was observed after the heating of the starting [PW_12_O_40_]^3–^@NU-1000 material up to 120 °C, while the supercritical-CO_2_ drying of the parent compound led to the POM positioning in the MOF mesopores. The dislocation of the POM units in the MOF was identified using powder XRD, volumetric N_2_ sorption isotherm analyses and density functional theory (DFT) calculations for the determination of pore size distributions. In addition, a significant effect of the POM location in the MOF on the catalytic properties of the resulting POM@MOF material was indicated by the oxidation of 2-chloroethyl ethyl sulfide which is a simulant of the chemical warfare agent mustard gas. Remarkably, the material containing POM units in the mesopores showed a faster substrate conversion than the individual POM and MOF compounds and the material with POM-loaded micropores. This is likely due to the relatively easy access of sulfide to POM and MOF sites within the hybrid, and the synergy between the two components of the assembly.^40^

**Fig. 4:**
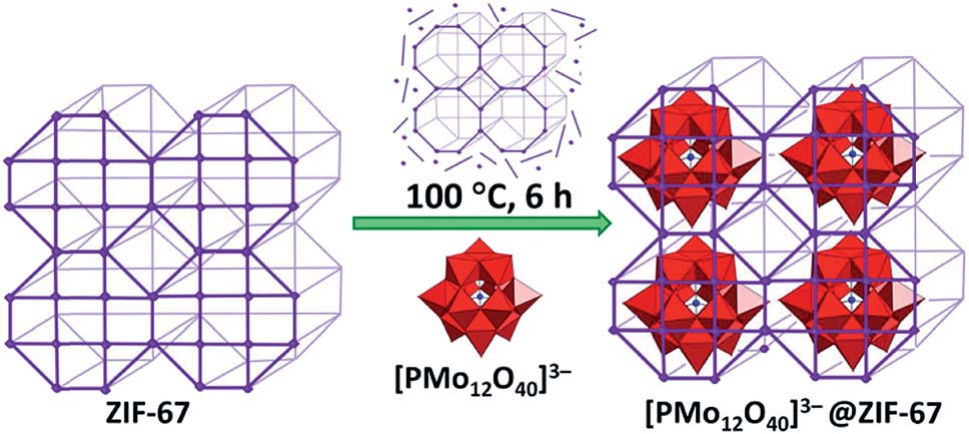
Schematic depiction of the synthetic procedure used for the preparation of the hybrid [PMo_12_O_40_]^3–^@ZIF-67. Colour code: W, red polyhedra; P, blue spheres. Adapted from ref. 41, with permission from The Royal Society of Chemistry.

Last but not least, the controlled assembly of POMs and MOFs towards the incorporation of the former into MOF cages that have windows smaller in size than the minimum POM dimension^35^,^41^ is actively being explored. Although the POM units in the visualised hybrids are supposed to be tightly included in the MOF cages, there is no need for their covalent interaction which may eliminate or negatively affect the valuable properties of both components.^13^ Thus, the advantages of the proposed innovative design of POM@MOFs may even overplay the covalent bonding approach, which is traditionally assumed to result in more stable compounds of POMs with MOFs than their supramolecular assemblies. This concept is particularly significant for the catalytically applicable POMs, as the integration of POMs in MOF pores with small openings is expected to prevent POM migration within or out of the MOF under catalytic reaction conditions. Furthermore, it should simultaneously grant access of the reactants to the POM active sites. The successful implementation of the approach was revealed by a few POM@MOF assemblies where [Co^II^Co^III^W_11_O_39_(H_2_O)]^7–^ or [Co_4_(PW_9_O_34_)_2_(H_2_O)_2_]^10–^ POMs were blocked inside cavities of the iron(iii)-containing MOF, abbreviated as MIL-100. The materials were obtained by the hydrothermal reaction of the respective, pre-synthesised POMs with compounds containing MOF fragments. The hybrids showed photocatalytic activity for water oxidation. In addition, the stability of these POM@MOFs under certain catalytic reaction conditions was indicated by the lack of leaching of POM units from the MOF cavities.35*a* The [PMo_12_O_40_]^3–^@ZIF-67 hybrid also features such a locking of POM units in the MOF cavities. The POM@MOF was prepared by the solvothermal reaction of a pre-synthesised ZIF-67 MOF with the [PMo_12_O_40_]^3–^ POM. During treatment the MOF undergoes partial decomposition, thus allowing penetration of POM units into the MOF cavities. The process of [PMo_12_O_40_]^3–^@ZIF-67 formation is completed by MOF self-restoration (Fig. 4).^41^

Overall, filling MOF cavities with POMs is beneficial to the characteristics of such hybrid materials that are today predominately studied for utilisation in catalysis.^8^,^9^,^11^,^19^,^31^,^35^,^40^,^42^ Several research studies were further devoted to the investigation of the potential of POM@MOFs to be applied in the areas of magnetism,^28^ gas adsorption,^36^ wastewater treatment^26^ and electrochemical energy conversion and storage.^33^,^37^ POM@MOFs have been shown to exhibit more advanced characteristics over bulk POM compounds. This is due to the fact that MOFs serve as excellent solid supports for the dispersion of POM-based species, increasing the surface area of the latter.

For instance, the compound [Zr_6_O_16_H_18_][TCPPH_2_]_2_[P_2_W_18_Co_4_]_0.2_·26H_2_O (TCPPH_2_ = tetrakis(4-carboxyphenyl)porphyrin), where the sandwich-type polyanions [(PW_9_O_34_)_2_Co_4_(H_2_O)_2_]^10–^ as catalysts are encapsulated in the pores of the cationic MOF-545 as a photosensitiser, is one of the recent successful illustrations of POM@MOFs showing high photocatalytic activity for visible-light-driven water oxidation.^19^ The oxidation of alkylbenzenes can, in turn, be realised with POM@MOFs obtained by dispersion of [PMo_12_O_40_]^3–^ or [SiMo_12_O_40_]^4–^ POMs in the cages of a three-dimensional (3D) [Cu_6_O(TZI)_3_(H_2_O)_9_]^+^ MOF (H_3_TZI = 5-tetrazolylisophthalic acid)26*b* or between the cationic two-dimensional (2D) lamellar networks as in {[Cd(DMF)_2_Mn^III^(DMF)_2_TPyP](PW_12_O_40_)}·2DMF·5H_2_O (DMF = *N*,*N*-dimethylformamide; TPyP = tetrapyridylporphyrin) (see Fig. 2c).26*a* Moreover, the hybrid materials of both these types exhibit highly efficient dye adsorption ability.^26^

In addition to the direct application as catalysts, POM@MOFs were also exploited as precursor materials for catalyst preparation. For instance, [PMo_12_O_40_]^3–^@ZIF-67 was used as the starting material for the synthesis of bi-transition metal carbide nanoparticles Mo_*x*_Co_*x*_C. The produced species showed sufficient electrocatalytic activity towards both the hydrogen and oxygen evolution reactions in a 1 M KOH aqueous solution.^41^

The supramolecular assemblies benefitting from the combination of intrinsic molecular attributes of POMs and MOFs for other application (in addition to catalysis) are also available.^28^,^33^,^36^,^37^ The grafting of the [(FeW_9_O_34_)_2_Fe_4_(H_2_O)_2_]^10–^ POM ({Fe_6_W_18_}) with single-molecule magnet (SMM) characteristics in diamagnetic or antiferromagnetic MOFs, that serve as a surface support, was performed in order to assess the dependence of POM magnetic properties on the type of MOF hosts used. It was demonstrated that the SMM behaviour of {Fe_6_W_18_} did not change after the latter was non-covalently implemented in diamagnetic UiO-67. By contrast, the antiferromagnetic MIL-101(Cr) framework eliminated the pristine SMM properties of the encapsulated {Fe_6_W_18_} due to the magnetic interactions between the MOF matrix and POM units.^28^ The utilisation of POM@MOFs towards the development of proton conductors was successfully demonstrated by incorporation of the Keggin-type POM [H_3_PW_12_O_40_] in cages of the MIL-101 MOF, followed by further modification of the [H_3_PW_12_O_40_]@MIL-101 hybrid with triethylenetetramine. The resulting material exhibits a proton conductivity value (1.52 × 10^–2^ S cm^–1^) close to the best reported value for individual MOFs.^33^ The compound H_3_[(Cu_4_Cl)_3_(BTC)_8_]_2_[PW_12_O_40_]_3_(C_4_H_12_N)_6_·3H_2_O (labelled NENU-11; BTC = 1,3,5-benzenetricarboxylate) in Fig. 2d has the ability to adsorb and decompose (through a hydrolysis reaction) a nerve gas simulant such as dimethyl methylphosphonate (DMMP), and the POM units play a major role in this.^36^ In addition, the electrochemical performance of a host–guest [Ag_5_(pyttz)_3_·Cl·(H_2_O)][H_3_SiMo_12_O_40_]·3H_2_O compound (pyttz = 3-(pyrid-4-yl)-5-(1*H*-1,2,4-triazol-3-yl)-1,2,4-triazolyl) as the anode material for lithium ion batteries illustrates the highly promising potential of POM@MOFs for energy storage.^37^

### 2.2 POMs merged with cationic metal complexes

The hybrid materials composed of non-covalently bound POMs and metal complexes are nowadays accessible to a wide range of different areas of application such as catalysis, wastewater purification and detection of volatile organic compounds.

The ability of large cationic rings [Cu_4_(bpp)_4_]^4+^ (bpp = 1,3-di-4-pyridylpropane) to enclose [β-As_8_V_14_O_42_(H_2_O)]^4–^ polyanions was observed in the [Cu_4_(bpp)_4_][β-As_8_V_14_O_42_(H_2_O)] compound which is a product of a solvothermal reaction. This compound could be employed in wastewater treatment due to its catalytic activity towards reduction of water polluting toxic Cr^VI^ ions.^43^

**Fig. 5:**
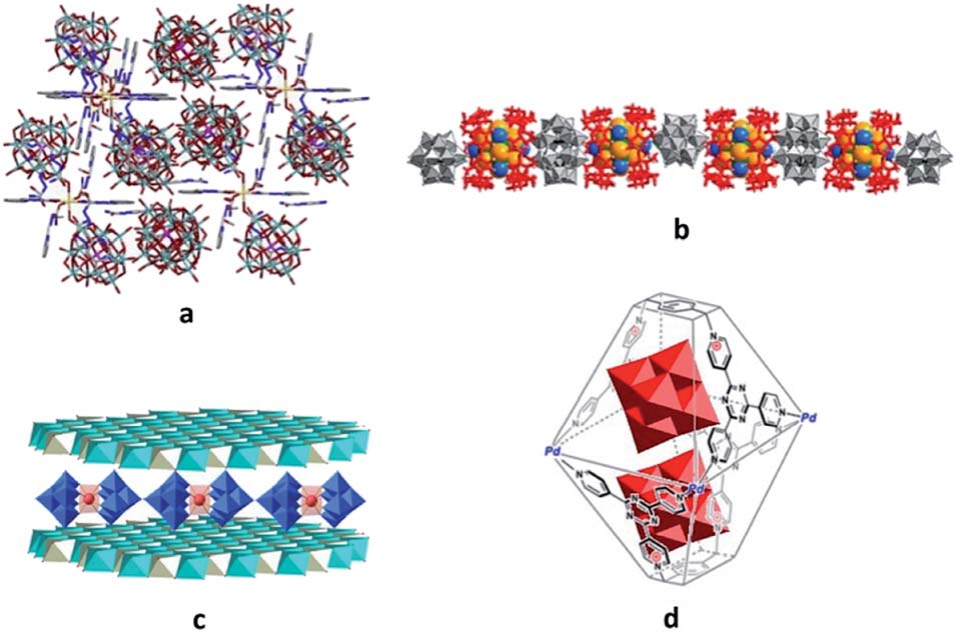
Polyhedral and/or ball-and-stick representation of different structural types of the assemblies and their segments constructed with POM and metal complex units. (a) {[Cd(H_2_L)_6_][H_2_L]_4_[PMo_12_O_40_]_4_}. Colour code: Mo, cyan; P, pink; Cd, yellow; N, blue; C, grey; O, red spheres. Reprinted from ref. 44, Copyright 2017, with permission from Elsevier. (b) {[Ta_6_Br_12_(H_2_O)_6_@2(γ-CD)][P_2_W_18_O_62_]}^4–^ chain. Colour code: W, red polyhedra; Ta, blue; Br, orange spheres. Reprinted with permission from ref. 45. Copyright 2011 American Chemical Society. (c) [EuW_10_O_36_]^9–^@[Mg_0.77_Al_0.23_(OH)_2_]^0.23+^. Colour code: W, blue; Al, cyan; Mg, gray polyhedra; Eu, red spheres. Reprinted from ref. 46*a*, Copyright 2017, with permission from Elsevier. (d) [Mo_6_O_19_]^2–^@[Pd_4_L_2_]^12+^. Colour code: W, red polyhedra. Adapted with permission from ref. 25. Copyright 2018 American Chemical Society.

Another highly promising environmental cleanup material is the sonochemically formed [Cd(H_2_L)_6_][H_2_L]_4_[PMo_12_O_40_]_4_·18CH_3_OH·4H_2_O compound where HL is pyridine-2-carbaldehyde semicarbazone (Fig. 5a). The assembly components ([Cd(H_2_L)_6_]^8+^, [H_2_L]^+^ and [PMo_12_O_40_]^3–^) interact with each other electrostatically as well as through hydrogen bonds. The hybrid has revealed high adsorption capacity to remove cationic dyes (methylene blue, rhodamine B) from aqueous solutions.^44^

The non-covalent assembly of [P_2_W_18_O_62_]^6–^ units and pre-synthesised host–guest cationic hybrids {Ta_6_Br_12_(H_2_O)_6_@2(γ-CD)}^2+^ (γ-CD = γ-cyclodextrin, C_48_H_80_O_40_) was performed by simply mixing aqueous solutions of both components under ambient conditions to yield K_2_H_2_{[Ta_6_Br_12_(H_2_O)_6_@2(γ-CD)][P_2_W_18_O_62_]}·32.5H_2_O. In the obtained compound the electrostatic forces and hydrogen-bonding between terminal oxygens of the POM units and aqua ligands of the {Ta_6_Br_12_(H_2_O)_6_}^2+^ facilitate the arrangement of the three-component {[Ta_6_Br_12_(H_2_O)_6_@2(γ-CD)][P_2_W_18_O_62_]}^4–^ fragments in the 1D chain-like structure (Fig. 5b). This hybrid assembly illustrates the competitiveness of the supramolecular approach as one of the adjustable routes for the synthesis of compounds with the desired composition of building block units and with a well-ordered structure.^45^

Several compounds resulting from the non-covalent incorporation of POM units between the positively charged sheets of the layered double hydroxides [M^II^_1–*x*_M^III^_*x*_(OH)_2_]^*x*+^[(A^*n*–^)_*x*/*n*_]^*x*–^·*y*H_2_O (M^II^ = Mg, Zn, Ni, *etc.*; M^III^ = Al, Cr, *etc.*; A^*n*–^ = Cl^–^, NO^3–^, CO_3_^2–^, *etc.*; *x* = M^II^/M^III^) were reported. The formation of assemblies of this type commonly occurs when the A^*n*–^ anions are exchanged for POMs. As an example, the structure of [EuW_10_O_36_]^9–^@[Mg_0.77_Al_0.23_(OH)_2_]^0.23+^ is shown in Fig. 5c. These hybrids trigger interest in their catalytic application due to the specific and precisely adjustable structures as well as the overall enhanced stability and catalytic activity compared to the parent compounds.^46^

The hybrid assemblies generated by the interaction of noble-metal based complexes with polyanions are described in the following. The self-assembly of positively charged container-molecules [Pd_4_L_2_]^12+^ (L = [*p*-xylene][2,4,6-tri-4-pyridyl-1,3,5-triazine]_2_) and POMs such as [Mo_6_O_19_]^2–^ or [Mo_8_O_26_]^4–^ resulted in the host–guest POM@[Pd_4_L_2_]^12+^ hybrids (Fig. 5d). Their formation process could be monitored using ^1^H NMR titration experiments, which were performed by simple addition of the guest POM compounds to the solution of the host metal complex. The resulting assemblies are likely generated due to electrostatic and anion–π interactions between the cage-shaping pyridinium units and POM anions. The improved catalytic performance of these POM@[Pd_4_L_2_]^12+^ hybrids in sulfoxidation reactions as compared to the activity of the pristine POM and the cage components emphasises the important role of the synergetic effects taking place in the supramolecular assemblies.^25^

Recently, it was shown that the non-covalent association of POMs ([W_6_O_19_]^2–^ and [α-Mo_8_O_26_]^4–^) with [Ir^III^(ppy)_2_(bpy)]^+^ (ppy = 2-phenylpyridine, bpy = 2,2′-bipyridine) can be achieved by mixing solutions of the corresponding ingredients under controlled conditions such as temperature, concentration of the reagents and reaction time. It was demonstrated that the electrostatic binding of POMs with [Ir^III^(ppy)_2_(bpy)]^+^ can significantly alter the solid-state phosphorescence of the latter depending on the structural type of the formative POM and the framework of the resulting hybrids. In addition, the simple preparation procedure and the revealed vapoluminescence properties of these hybrids make them suitable candidates to serve as sensors for the selective detection of volatile organic compounds.^47^

The networking of POM units and positively charged silver(i)-containing moieties led, in several cases, to the formation of supramolecular assemblies with different dimensions, *e.g.* a 2D {[Ag(DMSO)_2_][(Ag_3_(DMSO)_6_)(H_2_V_10_O_28_)]·2DMSO}_*n*_ (DMSO = dimethyl sulfoxide)48*a* and a 1D chain-like HNa_2_[(3-pya)(3-Hpya)Ag]_2_[AlMo_6_H_6_O_24_]·8H_2_O (3-Hpya = 3-(3-pyridyl)acrylic acid).48*b* These hybrids were obtained by mixing solutions of the corresponding standard salts and precursors under controlled conditions such as temperature, concentration of the reagents and reaction time. Exemplarily, the former was formed using AgNO_3_ and TBA_3_[H_3_V_10_O_28_] (TBA = tetrabutylammonium). In both cases, however, weak coordinative bonding between POM and Ag(i)-based species comes into play along with non-covalent interactions.

### 2.3 Architectures generated by solely POM units

The family of supramolecular assemblies that are predominantly constructed from POM units can be subdivided into two groups: (i) POM@POM hybrids formed due to the host–guest interactions between polyoxoanions of two different types27a,^49^,^50^ and (ii) self-assembled POM nanostructures with diverse dimensions.

**Fig. 6:**
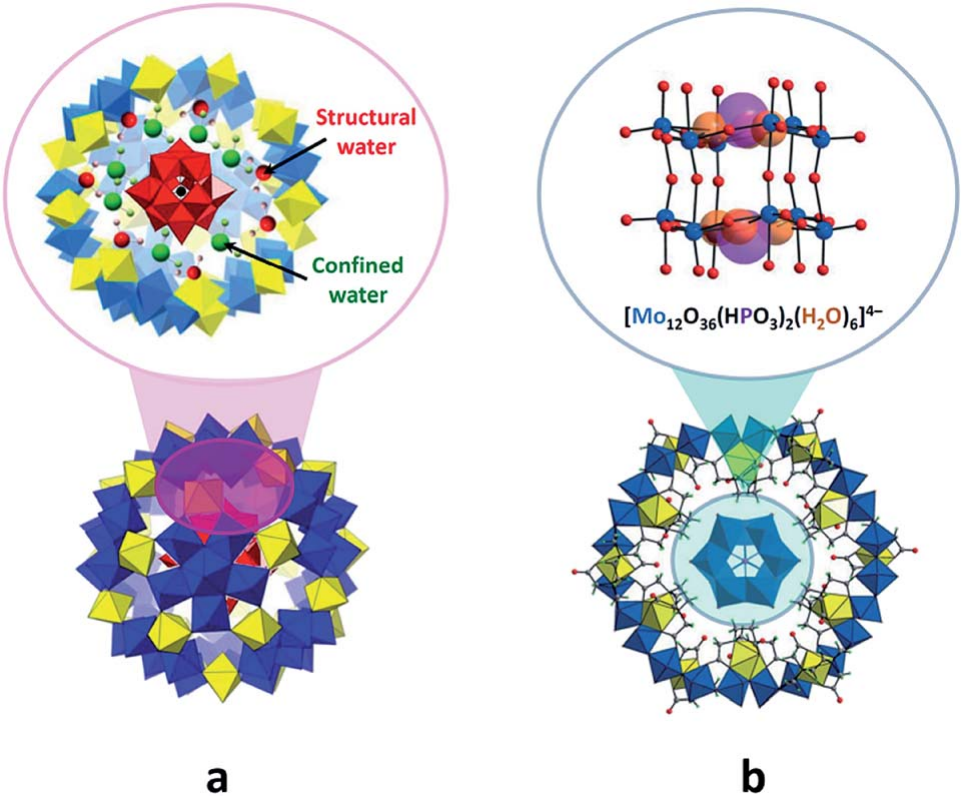
Polyhedral and ball-and-stick representation of assemblies formed by POM units. (a) {SiMo^VI^_12_O_40_@Mo_72_Fe_30_}, emphasizing the water molecules stabilizing the core–shell architecture. Adapted with permission from ref. 49*c*. Copyright 2016 American Chemical Society. (b) {(Mo_12_O_36_(HPO_3_)_2_(H_2_O)_6_)@Mo_24_Fe_12_}, showing the internal [Mo_12_O_36_(HPO_3_)_2_(H_2_O)_6_]^4–^ fragment structure. H atoms are omitted. Adapted from ref. 27*a*. Copyright 2016 of Wiley-VCH Verlag GmbH & Co. KGaA. Colour code: Mo, blue/red polyhedra or blue spheres; Fe, yellow polyhedra; P, violet; Si, black; C, gray; H, light green/pink; O, orange/green/red spheres.

The POM@POMs of the first group are generally synthesised *via* one-pot reactions, in which the pre-synthesised guest POM is utilised as a template, reacting with the substances consisting of host POM components.^49^,^50^ The POM@POMs, where the giant spherical {Mo^VI^_68_Mo^V^_4_Fe^III^_30_O_252_(CH_3_CO_2_)_16_(H_2_O)_100_} (abbreviated as Mo_72_Fe_30_) Keplerate-POM hosts Keggin-type polyanions such as [XM^VI^_12_O_40_]^*n*–^ (X = B^III^, Si^IV^, P^V^), represent the most studied examples.^49^ The formation mechanism of one of such assemblies, Na_6_[SiMo^VI^_12_O_40_@Mo_72_Fe_30_], in aqueous medium was elucidated by the solution-sensitive small-angle X-ray scattering (SAXS) and inelastic/quasi-elastic neutron scattering techniques, which revealed the presence of structural and confined water molecules along with the Keggin-guest species inside the Mo_72_Fe_30_ cavity. Moreover, the results of the studies showed that water molecules steer the position of the Keggin anion to the centre of Mo_72_Fe_30_ and stabilise the host–guest architecture. This finding emphasises the substantial role of water guests in the self-assembly process of compounds of the given type (Fig. 6a).49*c* In addition, it has been shown that differently sized and shaped POMs such as [SiMo_12_O_40_]^4–^ and [P_2_W_18_O_62_]^6–^ can be incorporated in the ring-like [Mo_24_Fe_12_(EDTA)_12_O_72_]^12–^ frameworks (abbreviated as Mo_24_Fe_12_) with EDTA = ethylenediaminetetraacetate.27a,^50^ The isolation of a Na_16_[(Mo_12_O_36_(HPO_3_)_2_(H_2_O)_6_)@Mo_24_Fe_12_]·85H_2_O compound (Fig. 6b) featuring the *in situ* generated [Mo_12_O_36_(HPO_3_)_2_(H_2_O)_6_]^4–^ species showcased the ability of the macrocyclic Mo_24_Fe_12_ to act as a “molecular reactor” for the synthesis of new kinds of polyoxoanions.27*a*

The second group of POM materials can be additionally subdivided into assemblies that exist in solution14a,^51^–^54^ and/or in the solid state.^55^ Here, some all-inorganic POMs as well as POMs covalently derivatised with organic moieties are capable of building supramolecular assemblies of different shapes and sizes in solutions under certain conditions when adjusting *e.g.* concentration, temperature and ionic strength.14a,^51^–^54^ The understanding and control of the self-assembly behaviour of POMs and their counterparts in liquid media are paramount because the configuration type of supramolecular architectures that are formed by solution processable, individual species may significantly alter the performance and efficiency of the generated compounds in application-related processes, *e.g.* in pharmaceutical experiments.

**Fig. 7:**
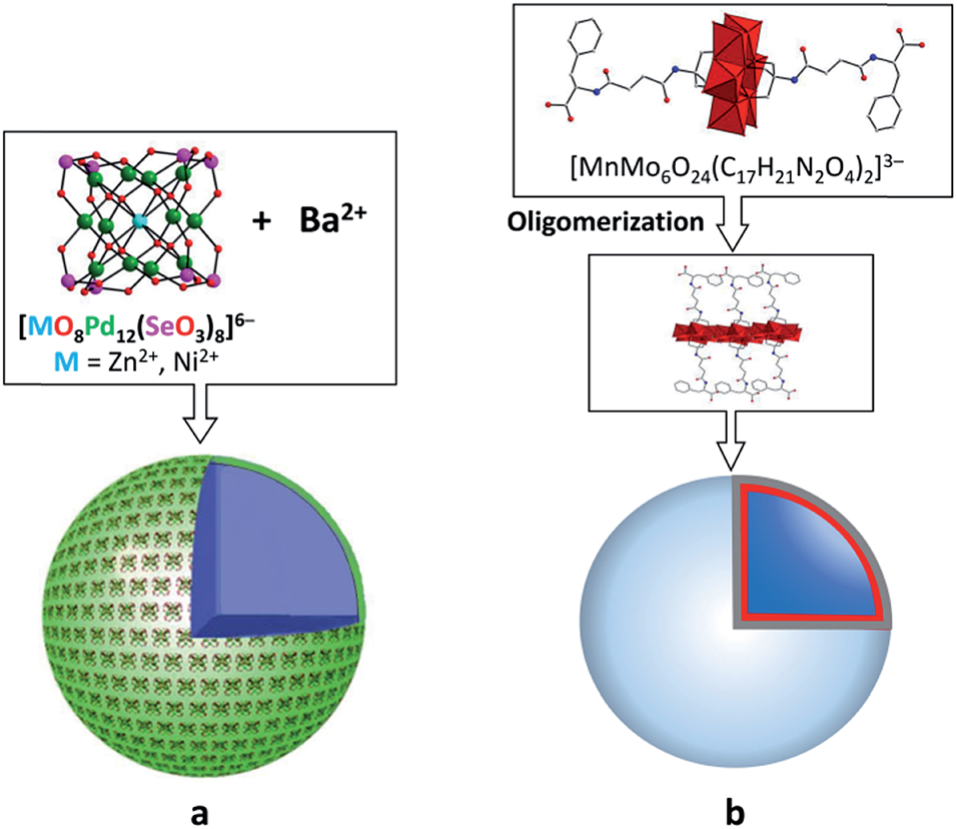
Schematic representation of the formation of “blackberries” by POM units. (a) Cubic-like [MO_8_Pd^II^_12_(Se^IV^O_3_)_8_]^6–^ in the presence of Ba^2+^ ions. Adapted with permission from ref. 52. Copyright 2018 of Wiley-VCH Verlag GmbH & Co. KGaA. (b) Anderson–Evans-type POM–peptide hybrids. Colour code: Mo, red polyhedra; N, blue; C, gray; O, red spheres. Adapted with permission from ref. 53*b*. Copyright 2018 of Wiley-VCH Verlag GmbH & Co. KGaA.

**Fig. 8:**
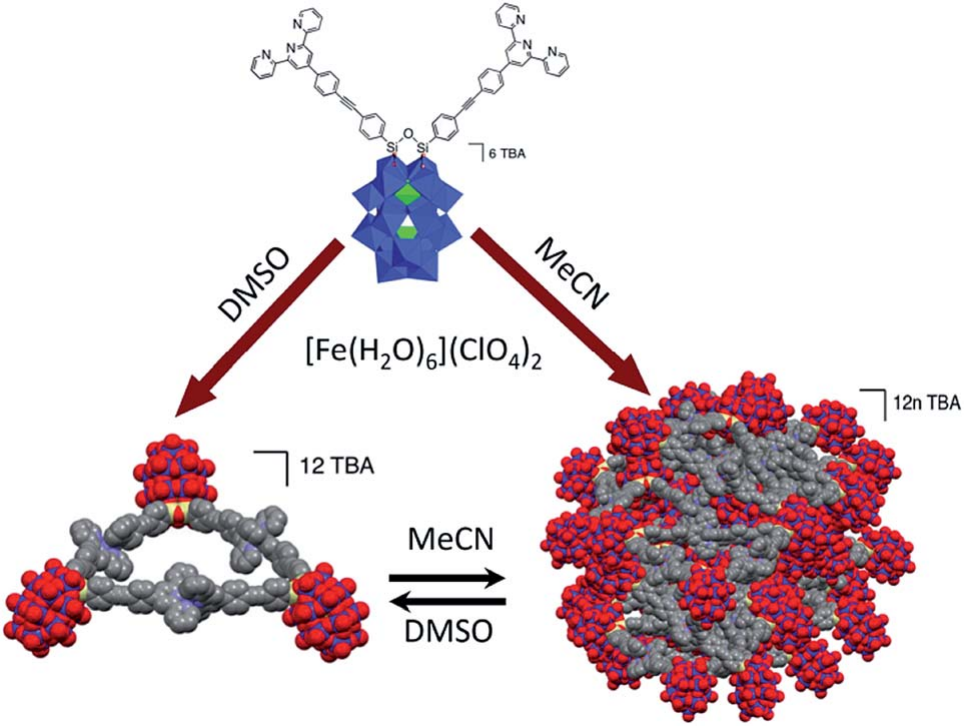
Schematic illustration of the formation of two types of nanostructures by complexation of TBA_6_[P_2_W_17_O_61_(O(Si–C_29_H_18_N_3_)_2_)]_3_ with Fe^2+^ ions. Colour code: W, blue polyhedra or spheres; P, green polyhedra; Si, yellow; N, violet; C, gray; O, red spheres. Adapted with permission from ref. 54. Copyright 2016 American Chemical Society.

The spherical single-layered nanostructures, so-called “blackberries” (Fig. 7a), that are formed by all-inorganic [MO_8_Pd^II^_12_(Se^IV^O_3_)_8_]^6–^ units (M = Zn^2+^, Ni^2+^) charged-balanced by Ba^2+^ or Sr^2+^ ions in water are examples of the POM supramolecular assemblies existing in solution.^52^ Recently, it was shown that POM–peptide hybrids are also able to form “blackberries” in the mixed acetonitrile/water solutions (Fig. 7b). The aggregation of the biomoiety-decorated POMs can be viewed as a model system for the investigation of various biomolecular interactions that may take place during biological processes.^53^ The diversity of the nanostructures self-assembled by POM-based supramolecular aggregates in various solvents was also demonstrated for the {[P_2_W_17_O_61_(O(Si–C_29_H_18_N_3_)_2_)]_3_·Fe_3_} compound which exhibits discrete triangular units in DMSO, while in acetonitrile the {[P_2_W_17_O_61_(O(Si–C_29_H_18_N_3_)_2_)]_3_·Fe_3_} moieties form monodisperse nanoparticles (Fig. 8).^54^

**Fig. 9:**
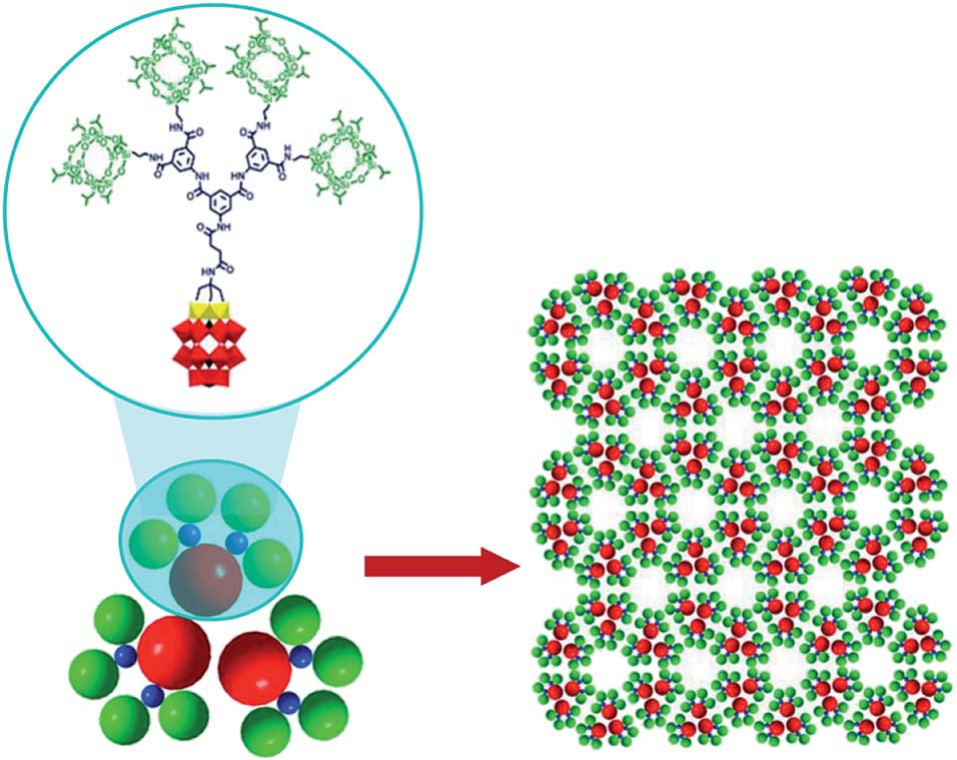
The honeycomb monolayer grid formed by (TBA)_6_[P_2_W_15_V_3_O_62_(POSS)_4_]. TBA ions are omitted for clarity. Colour code: W, red; V, yellow; P, black polyhedra. Adapted with permission from ref. 55*a*. Copyright 2018 American Chemical Society.

The existence of POM supramolecular assemblies in the solid state is indicated by a large diversity of well-ordered structures (chains, sheets, and porous materials) shaped by polyoxoanions, associated countercations (usually alkali metal or alkylammonium ions) and water molecules *via* supramolecular interactions (mostly electrostatic). The (TBA)_6_[P_2_W_15_V_3_O_62_(POSS)_4_] compound with POSS = polyhedral-oligomeric-silsesquioxane exhibits one of these remarkable solid-state structures where the POM units are arranged in a honeycomb monolayer grid (Fig. 9). The network is generated due to two types of supramolecular interactions: the electrostatic interplay between the POM and TBA ions leads to the formation of trimers as shown in Fig. 9, while van der Waals forces between the POSS units of the polyanions link the formed trimers together. The resulting hybrid architecture can be seen as self-assembled artificial graphene.55*a*

### 2.4 POMs assembled with organic molecular architectures

The incorporation of POMs into organic matrices *via* non-covalent interactions usually results in the formation of 2D^15^ and 3D materials with variable properties, which have considerable application potential as *e.g.* energy storage materials^10^ or anticancer drugs.^5^,20c,^56^ The compounds of this type available so far can be subdivided into several groups depending on the type of organic constituents such as polymers, lipid-based derivatives, cyclodextrins and organic cations.

The polymers host POM units in numerous hybrid assemblies.^13^,14a,20a,c,^28^,^56^–^69^ The non-covalent combination of POMs with polymers is usually realised through mixing of solutions of POMs and polymers under controlled reaction conditions (pH, temperature, stirring time and concentrations of the reagents), which leads to the separation of the resulting POM@polymer hybrids in the form of a liquid phase (coacervate).^13^,14a,20c,^28^,^57^–^60^,^62^–^66^,^68^ However, other synthetic procedures for the preparation of these hybrid materials were also explored. For instance, a reversed-phase microemulsion polymerisation method was used to generate POM@starch nanoparticles.^56^ The incorporation of [NaP_5_W_30_O_110_]^14–^ polyoxoanions through a metathesis reaction (exchange with PF_6_^–^ counteranions) into a positively charged poly-zinc-octaethylporphyrin/viologen-based polymer (poly-ZnOEP) film, which was pre-fabricated on an indium-tin-oxide (ITO) electrode, yielded a [NaP_5_W_30_O_110_]^14–^@poly-ZnOEP hybrid.^67^ Notably, the layer-by-layer deposition was recently utilised for the preparation of POM@polymer materials.^61^,^69^

The electrostatic interaction between positively charged polymer terminals and polyoxoanions is frequently utilised to synthesise the hybrids.^13^,14a,20a,c,^28^,^57^–^65^,^67^–^69^ Physical absorption,^56^ host–guest interactions^61^ and hydrogen bonding^66^ are also known to be driving forces for the association processes towards POM@polymer hybrids.

Natural polymers such as starch,^56^ gelatin^28^,^57^,^58^ and chitosan20c,^59^ are in the focus mostly due to their ability to serve as drug carriers. It was proven that assembling the above-mentioned natural polymers with bioactive POMs positively influences the biological characteristics (*e.g.* cytotoxicity and cellular uptake) of the resulting hybrid materials when compared with those of the corresponding pristine POM compounds.^5^,20c,^56^ Moreover, the association of POMs with natural polymers gives rise to novel materials with valuable mechanical,^57^ catalytic^58^ and photoluminescent^60^ properties.

The magnetic properties of the assemblies made of POMs and natural polymers were also assessed. The successful usage of gelatin (Gel) as a matrix for the encapsulation of the aforementioned Fe_6_W_18_-SMM [(FeW_9_O_34_)_2_Fe_4_(H_2_O)_2_]^10–^ (see Section 2.1) was reported. It was shown that the SMM properties of the bulk sodium-tetramethylammonium salt of this polyanion were preserved after the Fe_6_W_18_ units were incorporated in the Gel.^28^

As recently reviewed,20*a* the interaction of POM units with peptides affords hybrids that exhibit valuable features (*e.g.* enhanced biocompatibility as compared to that of the respective pristine POMs) for biological applications.

The host–guest and electrostatic interactions of bis-biotinylated POM [γ-SiW_10_O_36_{(C_5_H_7_N_2_OS)(CH_2_)_4_CONH(CH_2_)_3_Si}_2_O]^4–^ with avidin (a tetrameric protein capable of binding biotin), were used to produce a biomaterial that showed peroxidase-like catalytic activity. Moreover, films of the 2D POM@avidin network on a diamond crystal surface could be obtained by means of the layer-by-layer deposition method. This demonstrates feasibility of POM@protein film engineering which is essential for the further application-oriented development of POM@protein materials.^61^

The potential for assembly formation of synthetic polymers and POMs was also investigated. A hybrid material consisting of the Keggin-type [PW_12_O_40_]^3–^ POM and a cationic peptide-based polymer was prepared and the differences in the mechanism of antimicrobial activity of the pristine polymer and the POM@polymer hybrid were established. Interestingly, a synergetic effect resulting from assembling the two molecular building blocks was indicated by observed enhanced antimicrobial activity of the hybrid compared with the performance of the individual components.^62^

**Fig. 10:**
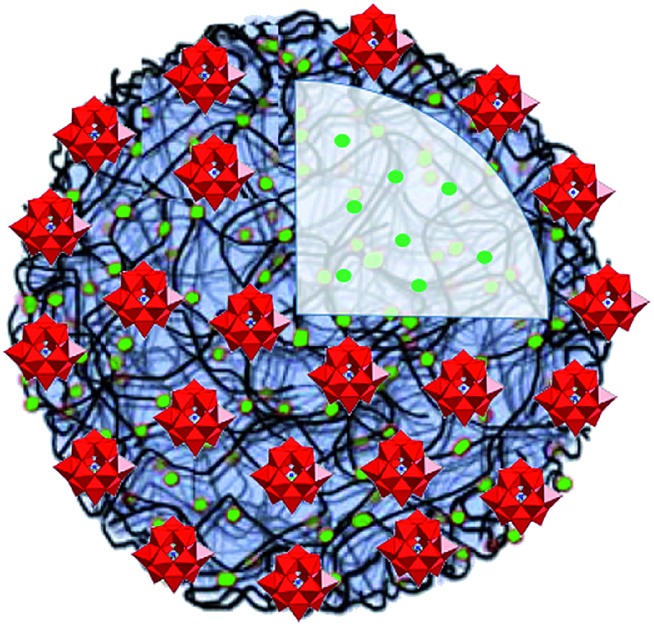
The POM coacervate vesicle formed by the Keggin-type [PW_11_O_39_]^7–^ units, ATP (green spheres) and PDDA (black bent lines). Colour code: W, red polyhedra; P, blue spheres. Adapted with permission from ref. 65. Copyright 2014 of Wiley-VCH Verlag GmbH & Co. KGaA.

A graphite electrode modified with a film of [PW_9_O_34_(*t*BuSiOH)_3_]^3–^-doped polycarbazole chains showed significant electrocatalytic activity for the amperometric detection of glucose, which makes these materials suitable for sensing applications.^63^ The association of the Anderson–Evans-type [MnMo_6_O_18_{(OCH_2_)_3_CNHC_21_H_19_N_2_O_4_}_2_]^3–^ polyoxoanions with cationic poly(2-(dimethylamino)ethyl methacrylate) yielded a material that in the solid state reveals high photosensitivity under low power UV-irradiation at room temperature.^64^ The POM coacervate vesicles (Fig. 10) shaped by [PW_11_O_39_]^7–^, adenosine-5′-triphosphate (ATP) and poly(diallyldimethyl ammonium chloride) (PDDA) were shown to exhibit cell-like properties.^65^

Embedding POMs into surface-supported polymeric porphyrin- and viologen-containing matrices has paved the way for photovoltaic devices. This approach can be exemplified by the significantly increased photocurrent response of the ITO electrode modified with the aforementioned [NaP_5_W_30_O_110_]^14–^@poly-ZnOEP film compared with that of ITO engineered with the POM-free poly-ZnOEP.^67^

Finally, the hybrid materials constructed from Cs_2.5_H_0.5_[PW_12_O_40_] in a polymethylsiloxane framework showed enhanced catalytic activity towards hydrolysis of ethyl acetate in water compared with the performance of the parent POM compound.^68^

Lipid-based compounds such as stearic acid56b,c or liposomes^70^ belong to another group of organics that are capable of forming compatible supramolecular assemblies with POMs. These POM@lipid hybrids can be obtained by reactions of (i) stearic acid with a POM compound (K_6_[SiMo_11_O_39_Co(H_2_O)]·*n*H_2_O or Na_5_[PMo_10_V_2_O_40_]·*n*H_2_O)56b,c and (ii) liposomes with K_6_[SiW_11_TiO_40_]·16H_2_O.^70^ Although the POM@liposome hybrids were shown to be formed due to physical absorption of the POM units on liposomes, stability tests indicated that the assemblies preserve the structural integrity only for 2 h in the buffer solution at physiological pH 7.4.^70^ Overall, the POM@lipid hybrids are characterised by higher antitumor activity than the activity of pristine POM compounds.56b,c,^70^

Cyclodextrins (CDs) – macrocyclic oligosaccharide molecules of different sizes (labelled α-, β- and γ-CDs consisting of 6, 7 and 8 glucose units, respectively) that are usually used for drug delivery^22^,27b,^46^,^71^–^74^ – have emerged today as reliable supramolecular carriers for POM units. The supramolecular assembly of POMs and CDs is generally performed using one-pot reactions, by mixing aqueous solutions of the corresponding ingredients under controlled conditions (pH, temperature and concentration of the reagents). It was shown in several studies that all-inorganic POM anions can be associated with CD molecules exclusively through intermolecular [C–H···O] contacts27b,^72^,^73^ or, in addition *via* electrostatic interactions and hydrogen bonds involving countercations that ultimately link CD and POM units together.^22^,^46^ POMs bearing at their molecular periphery structurally exposed aromatic groups also tend to form POM@CD host–guest complexes due to sustained non-covalent interactions between these organic units and the CD platform.^74^ The stability of POM@CD complexes in water was demonstrated in a number of studies.^22^,27b,^72^–^74^

Atypically for the aforementioned assemblies of POMs and organic molecules, the structures of the majority of all known POM@CD complexes could be determined *via* single-crystal XRD. This indicates the ability of CDs to serve as convenient agents for supramolecular POM crystallisation and, thus, offers an interesting strategy for the preparation of novel, structurally well-defined POM-based functional compounds and nanomaterials. As an example, the combination of [PVW_11_O_40_]^4–^, α-CD and cationic pseudorotaxane ([C_42_H_42_N_6_O_4_]^2+^) units in one assembly was found to be beneficial for size-selective separation of semiconductor quantum dots.^22^

**Fig. 11:**
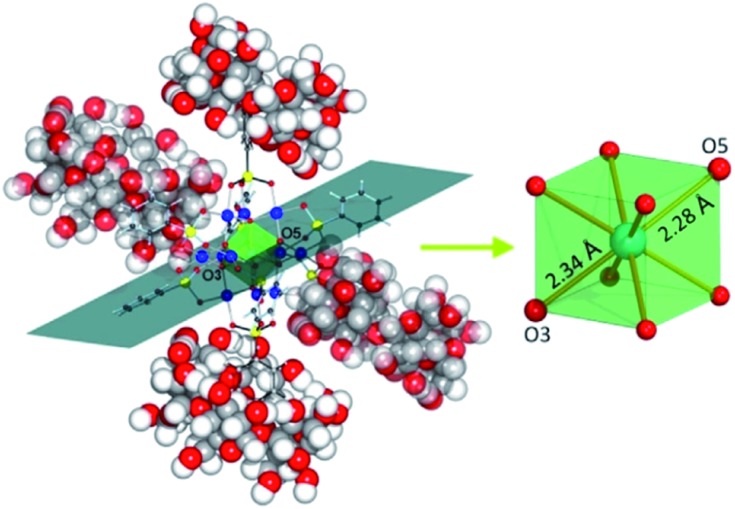
Representation of the fragment of the solid-state structure of K_6_[{CoO_8_Pd_12_(C_6_H_5_AsO_3_)_8_}⊂2{C_36_H_60_O_30_}]·25H_2_O·KCl, emphasizing the elongation of the {CoO_8_} unit (transparent green) along one of its diagonal planes. Bonds in the two α-CD-incorporated phenylarsonates are shown in black. Counterions, water and co-crystalised KCl molecules are omitted for clarity. Color code: Co, turquoise; Pd, blue; As, yellow; O, red; C, black/grey; H, white spheres. Reprinted with permission from ref. 74*c*. Copyright 2018 of Wiley-VCH Verlag GmbH & Co. KGaA.

Contrary to the commonly unaltered polyoxoanion skeletons in supramolecular assemblies, the structural changes of the POM were clearly observed using a single-crystal XRD experiment performed for the K_6_[{Co^II^O_8_Pd^II^_12_(C_6_H_5_As^V^O_3_)_8_}⊂2{C_36_H_60_O_30_}]·25H_2_O·KCl compound. Specifically, non-covalent interactions between the peripheral phenyl groups of the [CoO_8_Pd_12_(C_6_H_5_AsO_3_)_8_]^6–^ polyoxoanion and the α-CDs cause modification in the geometry of the central {CoO_8_} POM moiety. The nearly cubic {CoO_8_} as observed in the parent Na_6_[Co^II^O_8_Pd_12_(C_6_H_5_AsO_3_)_8_]·26H_2_O compound became disproportionately stretched along one of its diagonal planes in the POM@CD hybrid (Fig. 11). These structural changes induced by the host–guest interactions were further shown to influence the electronic configuration of Co^2+^ ions in the assembly.74*c*

**Fig. 12:**
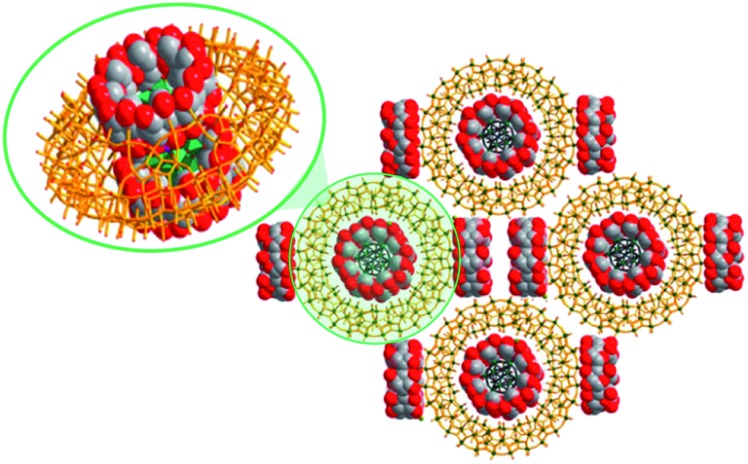
Packing in the solid-state structure of Na_15_K_3_H_2_[Mo_154_O_462_H_14_(H_2_O)_70_@P_2_W_18_O_62_@(γ-CD)_4_]·225H_2_O, emphasizing a {[P_2_W_18_O_62_]@2γ-CD@Mo_154_} segment. Counterions, water molecules and H ions are omitted for clarity. Color code: W, green polyhedra or spheres; Mo, dark green; O, red; C, grey spheres. Adapted with permission from ref. 73. Copyright 2018 American Chemical Society.

Remarkably, supramolecular POM@CD assemblies were themselves identified in the cavities of some POM molecules. As an example, the compound Na_15_K_3_H_2_[P_2_W_18_O_62_@(γ-CD)_4_@Mo_154_O_462_H_14_(H_2_O)_70_]·225H_2_O (Fig. 12) features a [P_2_W_18_O_62_]^6–^@2(γ-CD) fragment that is encapsulated in a central void of the giant wheel-shaped [Mo_154_O_462_H_14_(H_2_O)_70_]^14–^ polyanion (abbreviated as Mo_154_).^73^ The existence of this supra-assembly could be evidenced by 1D and 2D multinuclear (^1^H, and ^31^P) NMR spectroscopy in D_2_O solution. The three-component supra-assembly was also studied in the solid state, in particular by single-crystal XRD. The crystallographic analysis showed that the [P_2_W_18_O_62_@(γ-CD)_2_@Mo_154_]^20–^ hybrids are arranged in a porous superstructure with two additional γ-CD molecules per each [P_2_W_18_O_62_@(γ-CD)_2_@Mo_154_]^20–^ present in the holes. It should also be noted that an inclusion of individual [P_2_W_18_O_62_]^6–^ units in Mo_154_ was not observed. This finding illustrates the significance and the far-reaching opportunities of non-covalent interactions in the formation processes of multi-component assemblies.

Further developments for this type of supramolecular assemblies imply the unexplored association of organically pre-functionalised (chemically modified) CDs with POMs. Notably, different types of materials containing CD derivatised molecules were already synthesised, *e.g.* nanofibers and nanowebs were obtained from methyl-β-cyclodextrins.^75^ Moreover, the CD–MOF aggregates grown on modified glass surfaces^76^ showed the potential to be exploited as molecular platforms capable of incorporating functional POM units through non-covalent interactions. This showcases interesting application perspectives for the resulting supramolecular constructs, particularly in the area of switchable and sensory surfaces.

The electrostatic interaction of POMs with different types of cationic organic units in solutions under controlled conditions (concentrations of the reagents, pH, temperature, and reaction time) was shown to result in nanostructures of various forms and sizes and with different properties.20b,^23^,^77^–^83^ For instance, combining the [V_10_O_28_]^6–^ polyanions with 1-dodecylimidazolium (C_12_im) cations yielded a hybrid [C_3_H_4_N_2_(C_12_H_25_)]_4_[H_2_V_10_O_28_]·0.5C_2_H_5_OH compound that was found to exhibit anhydrous proton conductivity (4.6 × 10^–5^ S cm^–1^ at 373 K).^78^ The electrostatic interaction of long-chain quaternary ammonium cations with POMs led to supramolecular materials^23^,^79^,^80^ whose application areas range from protective coating for natural stones^23^ to water treatment.^80^ The aggregation of [SiW_12_O_40_]^4–^ with the cationic poly(amidoamine) (PAMAM) dendrimer of generation 4 (G4) afforded the POM–dendrimer hybrid material exhibiting photocatalytic activity towards degradation of an organic dye – methyl red in aqueous solutions – which is significantly enhanced compared with the activity of the parent POM compound K_4_[SiW_12_O_40_].^81^

The incorporation of POMs into cationic covalent organic frameworks could be carried out *e.g.* by the exchange of Br^–^ counterions of the covalent organic framework EB-COF (product of a Schiff base reaction of 3,8-diamino-5-ethyl-6-phenylphenanthridinium bromide with 1,3,5-triformylphloroglucinol) for the Keggin-type [PW_12_O_40_]^3–^ POMs. The resulting hybrid material [PW_12_O_40_]^3–^@EB-COF in which the POM units occupy the pores of the EB-COF, is thus characterised by significantly enhanced proton conductivity compared with that of the individual EB-COF at room temperature.^83^

The role of non-covalent interactions was also shown in the formation of POMs with relatively small organic moieties such as 4-amino pyridine (4-ap). The hybrid assemblies [H_8_(SiMo_12_O_40_)_2_][(C_5_H_6_N_2_)_*n*_]·7H_2_O (*n* = 9–10), [H_4_(SiMo_12_O_40_)][(C_5_H_6_N_2_)_6_] and [H_8_(SiMo_12_O_40_)_2_][(C_5_H_6_N_2_)_7_]·*x*H_2_O (*x* = 6–7) were prepared by hydrothermal treatment of a mixture of pyridyl naphthalene diamine and the compounds containing metal–oxo building block entities (namely, Na_2_MoO_4_·2H_2_O and Na_2_SiO_3_·5H_2_O). The 4-ap species were generated as a result of the pH dependent hydrolysis of pyridyl naphthalene diamine during the reaction. This method utilising *in situ* formed POMs and organic moieties for the preparation of POM-containing assemblies appears to be quite uncommon among the synthetic procedures for the compounds described in this section, where the pre-synthesised supramolecular assembly components are usually used. It was furthermore demonstrated that the adjustment of the reaction pH before hydrothermal treatment allowed the achievement of control of the POM/organic moiety ratio in the produced hybrids.^84^

Also the feasibility of implanting the Keggin-type POMs (H_3_PM_12_O_40_, M = Mo, W) into a caprolactam matrix was reported. It was shown that decreasing the POM/caprolactam ratio enforces transformation of the resulting hybrid material from the solid to the gel-like state.^85^

**Fig. 13:**
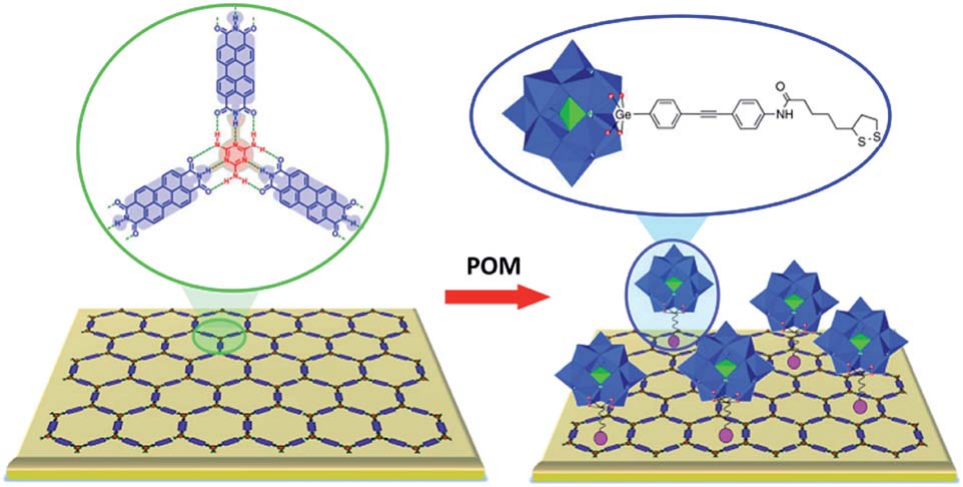
Schematic depiction of the organisational preferences of the Keggin-type POM-based inorganic–organic hybrid units [PW_11_O_39_Ge{*p*-C_6_H_4_–C≡C–C_6_H_4_–NHC(O)(CH_2_)_4_{–CH–(CH_2_)_2_S–S–}}]^4–^ (right) on the PTCDI/melamine-templated gold surface (left). Colour code: W, blue; P, green polyhedra; O, red spheres. Adapted with permission from ref. 29. Copyright 2016 American Chemical Society.

Overall, non-covalent interactions between organic molecules and POMs can be employed to attain a uniform arrangement of the metal–oxo cluster units on various substrate surfaces. Thus, POMs were found to be effectively attached onto surfaces of *e.g.* carbon nanotubes and graphene due to physisorption^24^,69a,^86^–^89^ or through linking moieties such as polymers^10^,^61^,^63^,^67^,69a or ionic liquids.^21^,^90^,^91^ The preparation of such POM-modified materials is usually performed either by simple impregnation of the substrates into solutions of the POM compounds^24^,69a,^86^–^88^,^90^ or by the layer-by-layer technique.^10^,^21^,^61^,^63^,^67^,69a,^90^,^91^ The molecular organisation of POMs on a gold surface *via* electrostatic interactions is best exemplified by the successful immobilisation of highly negatively charged [H_7_P_8_W_48_O_184_]^33–^ POMs onto the positive charge bearing 8-amino-1-octanethiol covered Au electrode.^92^ The host–guest interactions of POMs with supramolecular organic assemblies can also be used to effectively pattern POM units on pre-functionalised gold surfaces. It was shown that the open pores of a 2D supramolecular honeycomb network, that is formed on Au due to hydrogen bonds between perylene-3,4,9,10-tetracarboxylic acid diimide (PTCDI) and 1,3,5-triazine-2,4,6-triamine (melamine), may host the POM-based inorganic–organic TBA_4_[PW_11_O_39_Ge{*p*-C_6_H_4_–C≡C–C_6_H_4_–NHC(O) (CH_2_)_4_{–CH–(CH_2_)_2_S–S–}}] hybrids. The latter are fixed in the openings of the PTCDI/melamine network through Au–S (POM) bonds (Fig. 13).^29^

Devices which are built *via* non-covalent attachment of the POM units at surfaces are actively investigated for application in catalysis,^61^,69a,^87^,^89^ sensorics,^63^,69a molecular electronics,^92^ neuromorphic computing,^24^,^93^ proton conductors^88^ and energy storage and conversion.^10^,^21^,^67^,69a,^86^,^90^,^91^

Last but not least, the loading of POMs into polymerisable ionic liquid matrices was shown to produce materials suitable for 3D printing. The simple mixing of H_6_[P_2_W_17_O_61_(P(=O)C_6_H_5_OC_10_H_21_)_2_]·3C_4_H_9_NO (C_10_POM) with 3-butyl-1-vinylimidazolium bis(trifluoromethane)sulfonimide ([BVIM][NTf_2_]) yielded a C_10_POM@([BVIM][NTf_2_]) compound that was layer-by-layer photopolymerised using digital light processing 3D printing. This technique allows for the fabrication of a 1 cm^3^ object that has the form of a so-called “Schwarz P Surface Cube”. In addition, the observed photochromic properties (reversible reduction under visible light irradiation) of the printed sample reveal the successful transfer of photo and redox characteristics of the C_10_POM molecules into the macroscopic object.^94^

## 3 Conclusions and outlook

Insights provided herein into the emerging class of polymeric and discrete organic and inorganic assemblies with POM units embedded through supramolecular contacts allow the identification of diverse perspectives for these molecular conjugates in materials science, catalysis, medicine, and energy storage and technology. It should be noted that the general requirements for the formation of POM-based supramolecular hybrids are indeed the compatible dimensions of the interacting components and their solubility and stability under certain reaction conditions with the need for systematic exploration. The constructional principles of POM-loaded assemblies may help explore the possibilities of the formation of technologically relevant 2D networks on surfaces by means of supramolecular chemistry and nano-engineering.

## Conflicts of interest

There are no conflicts to declare.
